# Assessing the Potential Earthquake Precursory Information in ULF Magnetic Data Recorded in Kanto, Japan during 2000–2010: Distance and Magnitude Dependences

**DOI:** 10.3390/e22080859

**Published:** 2020-08-01

**Authors:** Peng Han, Jiancang Zhuang, Katsumi Hattori, Chieh-Hung Chen, Febty Febriani, Hongyan Chen, Chie Yoshino, Shuji Yoshida

**Affiliations:** 1 Department of Earth and Space Sciences, Southern University of Science and Technology, Shenzhen 518055, China; hanp@sustech.edu.cn (P.H.); 11930858@mail.sustech.edu.cn (H.C.); 2 The Institute of Statistical Mathematics, Tokyo 190-8562, Japan; zhuangjc@ism.ac.jp; 3 Graduate School of Science, Chiba University, Chiba 263-8522, Japan; chie@earth.s.chiba-u.ac.jp (C.Y.); shuji@faculty.chiba-u.jp (S.Y.); 4 Center for Environmental Remote Sensing, Chiba University, Chiba 263-8522, Japan; 5 Institute of Geophysics and Geomatics, China University of Geosciences, Wuhan 430074, China; zjq02010@cug.edu.cn; 6 State Key Laboratory of Geological Processes and Mineral Resources, China University of Geosciences, Wuhan 430074, China; 7 Research Center for Physics, Indonesian Institute of Sciences, Tangerang, Banten 12710, Indonesia; febty82@gmail.com

**Keywords:** ULF magnetic data, earthquake precursory information, Molchan’s error diagram, Kanto, Japan

## Abstract

In order to clarify ultra-low-frequency (ULF) seismomagnetic phenomena, a sensitive geomagnetic network was installed in Kanto, Japan since 2000. In previous studies, we have verified the correlation between ULF magnetic anomalies and local sizeable earthquakes. In this study, we use Molchan’s error diagram to evaluate the potential earthquake precursory information in the magnetic data recorded in Kanto, Japan during 2000–2010. We introduce the probability gain (*PG′*) and the probability difference (*D′*) to quantify the forecasting performance and to explore the optimal prediction parameters for a given ULF magnetic station. The results show that the earthquake predictions based on magnetic anomalies are significantly better than random guesses, indicating the magnetic data contain potential useful precursory information. Further investigations suggest that the prediction performance depends on the choices of the distance (*R*) and size of the target earthquake events (*Es*). Optimal *R* and *Es* are about (100 km, 10^8.75^) and (180 km, 10^8.75^) for Seikoshi (SKS) station in Izu and Kiyosumi (KYS) station in Boso, respectively.

## 1. Introduction

Electromagnetic perturbations prior to fault ruptures or volcanic eruptions have been intensively studied during the past decades [1,2,3,4,5,6,7,8,9,10,11,12,13,14,15]. Generally, these electromagnetic changes can be classified into two types: (1) perturbations in the atmosphere and the ionosphere; (2) electric and magnetic changes in the lithosphere. The latter are commonly recorded by passive ground-based observations [5,16]. The measurement of ultra-low-frequency (ULF: less than one Hertz) electromagnetic phenomena may be one of the most promising candidates, because of their deeper skin depths [1,2,8,17]. To date, a large number of ULF electromagnetic phenomena associated with earthquakes has been reported from different parts of the world, e.g., Greece [18,19,20,21,22,23], Italy [24,25], Russia [26,27]; America [28,29,30], Mexico [31,32,33,34], Taiwan [35,36,37,38], Indonesia [39,40], China [41,42,43,44,45,46,47] and Japan [48,49,50,51,52,53,54,55,56,57,58,59,60]. Furthermore, experiments and simulations have confirmed the existence of ULF seismo-electromagnetic phenomena [61,62,63,64,65,66,67,68,69,70,71].

Nevertheless, due to the difficulties and considerable costs in maintaining ULF magnetic observations, long-term monitoring of electromagnetic signals in a seismic area is quite rare. To date, most reports of seismomagnetic phenomena are case studies. To reveal general characteristics of electromagnetic signals associated with earthquakes, a long-term continuous observation network was installed in the Kanto region, Japan at the end of last century. Recently, Hattori et al. [72] analyzed the data and verified the correlation between ULF magnetic anomalies and local sizeable earthquakes by statistical studies. However, how these anomalies can improve the forecasting of sizable earthquakes has not been demonstrated clearly. On the other hand, previous studies suggest that the earthquake-related magnetic signals may decrease with the epicentral distance and increase with the size of earthquake event [8,27,41,73,74], which implies that augmenting earthquake forecasting with electromagnetic anomalies may involve distance and magnitude dependences. In this study, making use of long-term magnetic measurements from Kanto, Japan during 2000–2010 and applying Molchan’s error diagram [75,76], we attempt to explore the optimal parameters of distance and magnitude for earthquake forecasting in Izu and Boso.

## 2. Data Analysis

**Figure 1:**
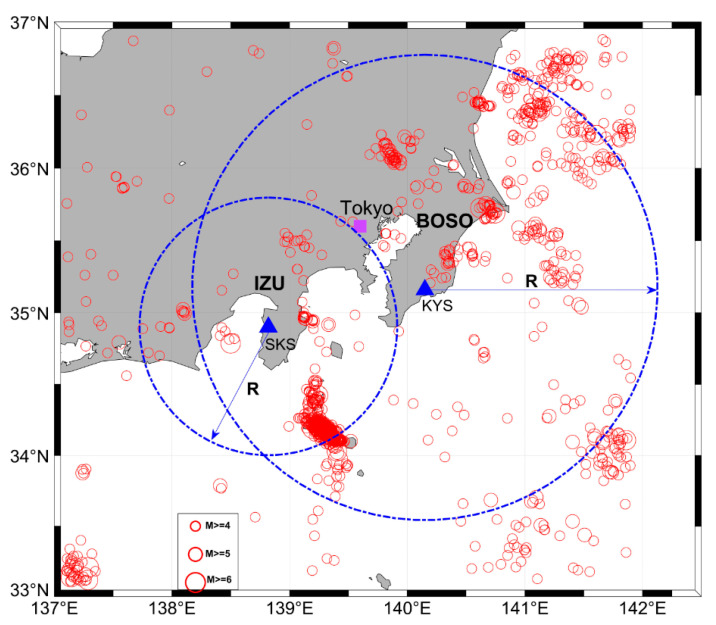
Spatial distribution of ultra-low-frequency (ULF) geomagnetic stations and nearby earthquake epicenters. Blue triangles indicate geomagnetic stations in Izu and Boso Peninsulas; red open circles present the epicenters of earthquakes with *M* ≥ 4.0 and *Depth* < 60 km during 2000–2010. Two blue circles show the distances of 180 km from Kiyosumi (KYS) and 100 km from Seikoshi (SKS), corresponding to the optimal distances *R* explored in Section 5.

In order to clarify ULF seismomagnetic phenomena, a sensitive geomagnetic network of torsion magnetometers with inter-station distances about 60 km was installed in the Kanto region, Japan. The details of the observation network are described in Hattori et al. [72]. Following that study, we choose to analyze the geomagnetic data observed at Seikoshi (SKS) and Kiyosumi (KYS) stations in Izu and Boso Peninsulas, respectively, because (1) seismic activities are intensive there; (2) the two stations contain the most complete data. Figure 1 shows their locations and epicenters of surrounding earthquakes with *M* ≥ 4.0 and *Depth* < 60 km during 2000–2010 based on the Japan Meteorological Agency (JMA) catalog.

The geomagnetic records consist of two orthogonal horizontal (N–S and E–W) and one vertical (Z) component, with a sampling rate of 1 Hz. Here, we focus on the Z component because it may include possible naturally enhanced energy signals prior to some large earthquakes [51,72,77,78,79]. The two horizontal components are also examined for reference. To minimize the influences of artificial noises, we use the data recorded only during 01:00–04:00 local time (LT) when the train system is shut down.

**Figure 2:**
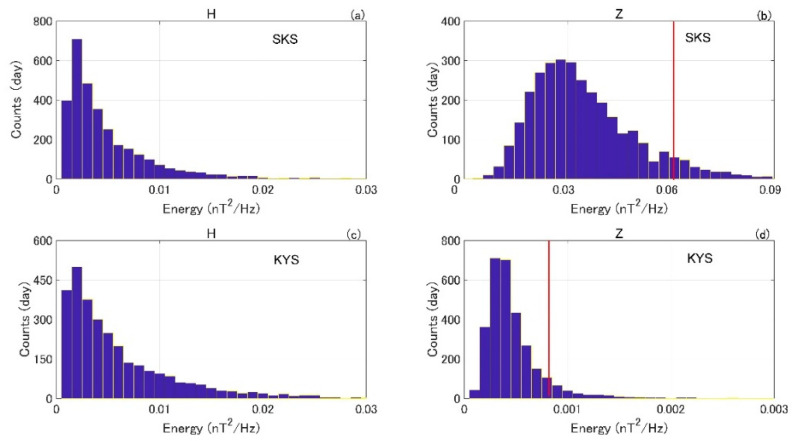
Histograms of energies during the years of 2000 to 2010. Panels (**a**,**b**) show results at SKS station in Izu; panels (**c**,**d**) show results at KYS station in Boso. Vertical red lines indicate the median +1.5 IQR threshold. The unit of energy in horizontal axis is nT^2^/Hz and the unit of counts in vertical axis is day (Modified after Hattori et al. [72]).

The same data analysis method as in Hattori et al. [51] is adopted. First, we apply wavelet transform to the 1-Hz nighttime geomagnetic data and extract the signals at the frequency around 0.01 Hz. Next, we compute the daily average energy of the obtained 0.01 Hz signals in the uncontaminated windows where there are no spike noises induced by sensor shaking during seismic event. Figure 2 plots the distribution of daily energies during 2000–2010 at each station. The total energy in horizontal component (H) is the sum of the energies in the N–S and E–W components. The energy variations in the H component at SKS and KYS are similar, while in Z component the variations are quite different. As the Z component is mainly the induction field, the differences between the two stations could be ascribed to variations of conductivity structures and/or electromagnetic environments. To identify global geomagnetic disturbances, the MMB station located in Hokkaido is taken as the reference.

## 3. Definition of ULF Magnetic Anomalies and Earthquake Events

The same as done in Hattori et al. [72], we define a magnetic anomaly when the energy of the Z component exceeds a certain threshold *P*. Considering that global magnetic storms may also lead to enhancement in the Z component, we exclude the anomaly when the energy of the H component in the reference station MMB exceeds median +3 IQR, where IQR is the interquartile range. The two red lines in Figure 2b,d indicate the *P* thresholds, which is the median +1.5 IQR value employed in previous superposed epoch analysis (SEA) in Hattori et al. [72]. Obviously, increasing (decreasing) the threshold *P* will give less (more) anomalies.

According to previous studies, whether an earthquake can produce observable signals at a given magnetic station depends on its magnitude and its distance to the station. Therefore, we use the *Es* parameter which is a function of the earthquake magnitude and the distance to define earthquake events [72]:

(1) $$Es=\underset{1day}{\sum }\frac{10^{4.8+1.5M}}{r^{2}}$$

where *M* and *r* are the magnitude of the earthquake and the hypocenter distance, respectively. The *Es* is daily sum of earthquake energies within the epicenter distance *R* from a magnetic station and its unit is J/km^2^. As only shallow events (*Depth* < 60 km) are taken into account, there is not much difference between hypocenter and epicenter distances, when *R* is larger than 80 km. Here we utilized *R* to select earthquakes to keep consistent with our previous studies. Following Hattori et al. [72], we define an event day when the *Es* parameter exceeds 10^8^ with *R* = 100 km for the SKS station in Izu and *R* = 150 km for the KYS station in Boso. In total, there are 60 event days for SKS in Izu and 92 event days for KYS in Boso, satisfying the criteria, respectively.

## 4. Assessing the Precursory Information in the Magnetic Data

### 4.1. Previous Statistical Results

Hattori et al. [72] applied SEA method to statistically investigate the distribution of magnetic anomalies relative to local sizable earthquakes in Izu and Boso Peninsulas. In order to evaluate the statistical significance, the distribution of magnetic anomalies relative to randomly selected days instead of earthquake days was also computed, that is the random_SEA for ULF anomaly at each station. They iterated the random_SEA test by 10,000 times and compute the mean (hereafter random_mean) and the corresponding standard deviation (σ).

Hattori and Han [74] summarized the results by SEA in Izu and Boso during 2000–2010. For comparison, the 5-day counts were normalized using the corresponding random_mean +2 σ values, which represent the statistical significances. Their results suggest a possible correlation between magnetic anomalies and earthquake events. In the following, we will investigate whether theses anomalies contain precursory information and how they can be used to improve the forecasting of earthquake events through Molchan’s error diagram. Particularly, we will examine the distance and magnitude dependences of precursory information in ULF magnetic data quantitatively.

### 4.2. Molchan’s Error Diagram

Since there may be a time lag between the precursory anomaly and the earthquake, Han et al. [80] introduced the leading time (Δ) and the alarm window (*L*) to specify the alarm function. In this study, we use the same alarm strategy as demonstrated in Hattori and Han [74]. The leading time (Δ) is set to 1 day and the alarm window (*L*) to 5 days. The information missing days are excluded in further analysis.

To evaluate the performance of the prediction algorithm, an “event day” is counted as “predicted” if it falls within an alarm interval or “missed” if it falls out of an alarm interval. The “detecting rate *ν*” and “alarm rate *τ*”, compute the ratio of predicted earthquake events and the ratio of alarmed days, respectively,

(2) $$\nu \left(P\right)=\frac{N_{1}}{N}$$

(3) $$\tau \left(P\right)=\frac{n_{1}}{n}$$

where *n* is the total number of days in the entire time period analyzed (excluding alarm missing days), *n*_1_ is the number of alarmed days, *N* is the total number of event days (excluding no prediction events) and *N*_1_ is the number of predicted event days [75,80,81]. *ν* and *τ* are functions of the threshold *P* which defines the magnetic anomaly. For a practical purpose, the alarm threshold *P* is set to change from the maximum to the minimum of the energy of the Z component. Correspondingly, *τ* and *ν* range from 0 to 1.

**Figure 3:**
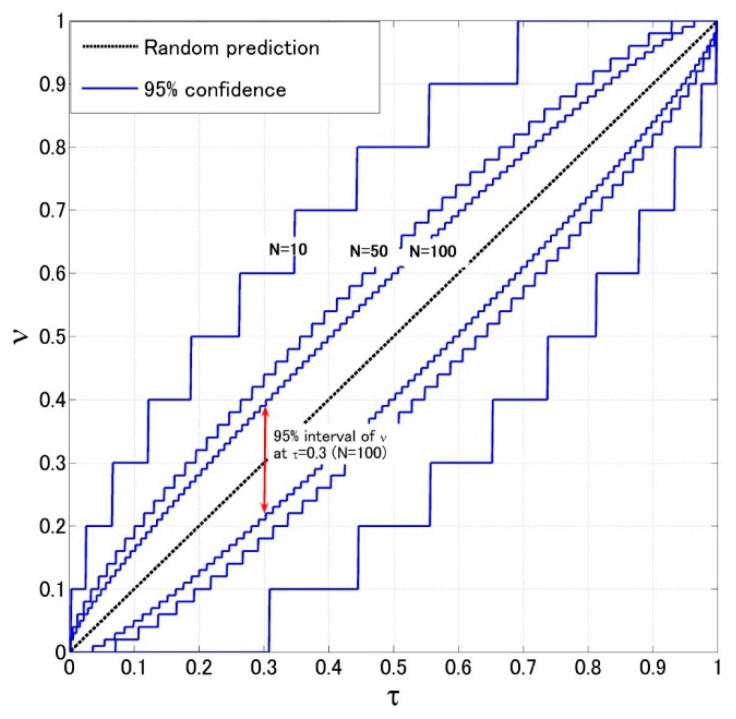
Molchan’s error diagram. The black diagonal line gives the most probable results of random guess; the blue lines present the 95% confidence intervals for different number of events (*N*). Red double arrow indicates the 95% interval of prediction at alarm rate *τ* = 0.3 and *N* = 100 using random guess.

The Molchan’s error diagram used here plots the detecting rate ν against the alarm rate *τ* (Figure 3), a little bit different from its original version which shows the unpredicted rate verse the alarm rate in Molchan [75]. In Figure 3, the extreme values with coordinates (0, 1) and (1, 0) are ideal and worst predictions, respectively. Generally, the detecting rate increases with the alarm rate. A diagonal line in the diagram indicates the prediction by the random guess (a Poisson model). Any prediction with *τ−ν* curve above this diagonal indicates the proportion of predicted earthquakes is greater than that of alarmed days, i.e., the prediction is better than the random guess. Otherwise, it is worse than the random guess. Actually, at a given alarm rate *τ*, the probability of *N*_1_ “detections” by using random guess follows a binomial distribution,

(4) $$B\left(N_{1}|N,\tau \right)=\left(\begin{array}{c} N\\ N_{1} \end{array}\right){\left(\tau \right)}^{N_{1}}{\left(1-\tau \right)}^{N-N_{1}}$$

As a result, one can compute the 95% confidence intervals of “detections” at each given alarm rate. In Figure 3, the 95% intervals are shown by blue lines for different *N*. The intervals get relatively larger when *N* becomes smaller, implying that the number of events (*N*) must be taken into consideration when accessing the forecasting performance.

### 4.3. The Efficiency of Magnetic Anomalies for Earthquake Forecasting

Hattori and Han [74] found that the prediction curves of both SKS and KYS are clearly better than the random guess and most of them exceed the 95% confidence threshold, suggesting the effectiveness of magnetic data in short-term earthquake forecasting.

For the practical application to short-term earthquake forecasting, we must choose one certain *P* threshold for issuing alarms. Hence, the actual prediction result should be only a signal point on the curve. A higher detecting rate causes more false alarms, whereas lower alarm rate causes more missing earthquakes. One useful index could be probability gain (*PG*), which is the ratio of the detecting rate *ν* to the alarm rate *τ* [74]. Another useful index could be the difference (*D*) between *ν* and *τ*. *PG* = 1 or *D* = 0 indicates completely random prediction.

**Figure 4:**
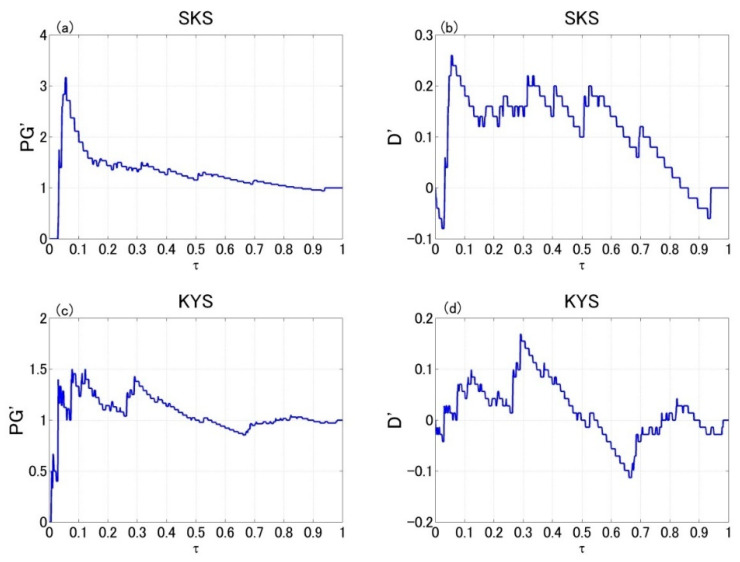
*PG′* and the *D′* indices for the predictions in Izu and Boso. (**a**,**b**) show the indices at SKS in Izu; (**c**,**d**) show the indices at KYS in Boso. *Es* and *R* parameters for earthquake selection are chosen the same as in Hattori and Han [74], namely *Es* = 10^8.0^, *R* = 100 km for SKS and *Es* = 10^8.0^, *R* = 150 km for KYS.

As seen in Figure 3, the confidence interval becomes larger when the event number gets smaller. Thus, it is unfair to access the forecasting performance using *PG* or *D* when *N* is not constant. To solve this problem, we introduce modified probability gain (*PG′*) and difference (*D′*) indices, which are computed as following:

(5) $$PG^{\prime }=\frac{\nu }{v_{up}}$$

(6) $$D^{\prime }=v-v_{up}$$

where *ν_up_* is the upper boundary of the confidence interval. Any prediction with *PG′* > 1 or *D′* > 0 is significantly better than random guess. Figure 4a,b show the *PG′* and *D′* indices of the prediction curve at SKS in Izu, respectively. Figure 4c,d show those at KYS in Boso. Here, the maximum *PG′* and *D′* exceeding the 95% confidence threshold are 3.17 and 0.26 at SKS, 1.5 and 0.17 at KYS, respectively. These predictions are significantly better than random guess, implying that the ULF magnetic data do contain precursory information of local sizable earthquakes and have potential capability to improve short-term earthquake forecasting.

## 5. Searching for Optimal Prediction Parameter

In previous section, we have showed the precursory information of the selected earthquake events by using Molchan’s error diagram. To date, the criteria for earthquake selection are based on empirical results. According to previous studies, the earthquake-related magnetic signals may decrease with the epicentral distance and increase with the size of earthquake event. As a result, earthquake events with large *R* or small *Es* may not create observable signals at the target magnetic station. A loose criterion (large *R* and small *Es*) can bring irrelevant events that do not produce magnetic anomalies and lead to increase of unpredicted cases, whereas a tight criterion (small *R* and large *Es*) can remove relevant events, which do generate magnetic anomalies and lead to increase of false alarms. The prediction curve could change significantly when using different *R* and *Es* thresholds. When approaching the optimal values of *R* and *Es*, the forecast efficiency increases. Otherwise, it decreases. Therefore, the prediction efficiency may exhibit *R* and *Es* dependences. In order to find out the most effective values, the parameter optimization analysis is applied.

**Figure 5:**
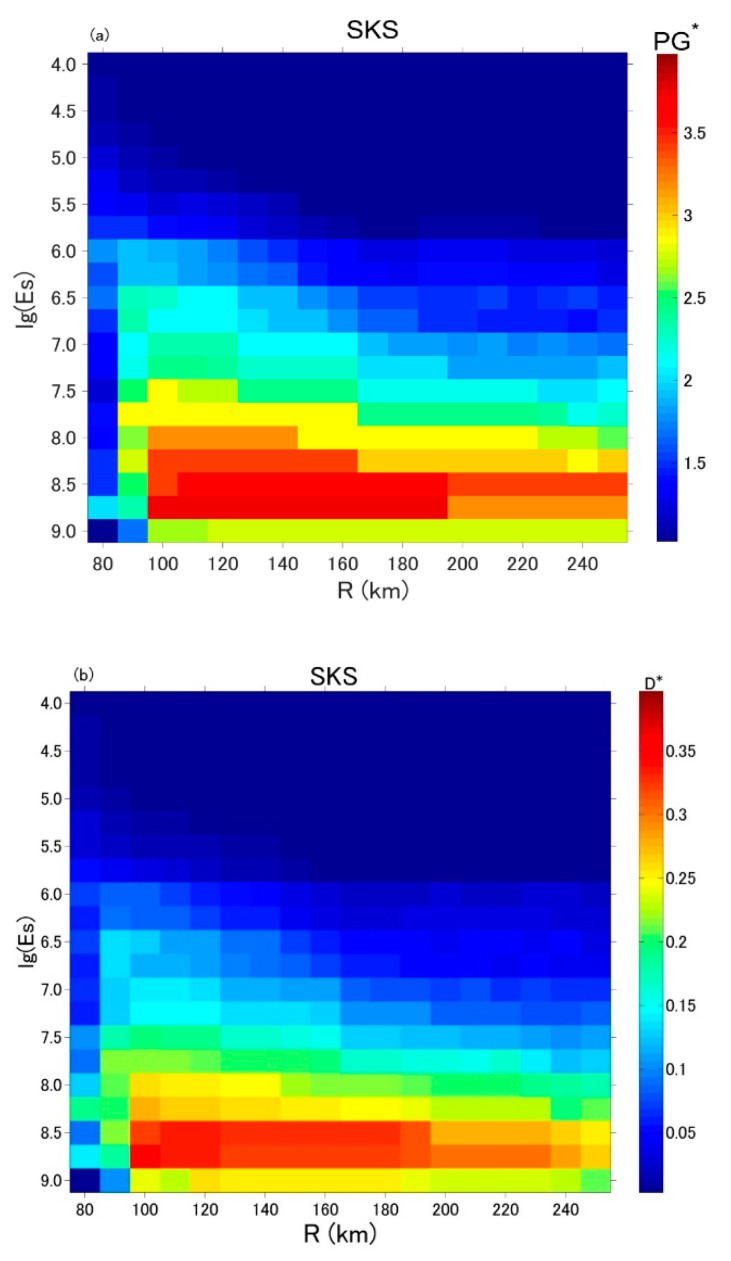

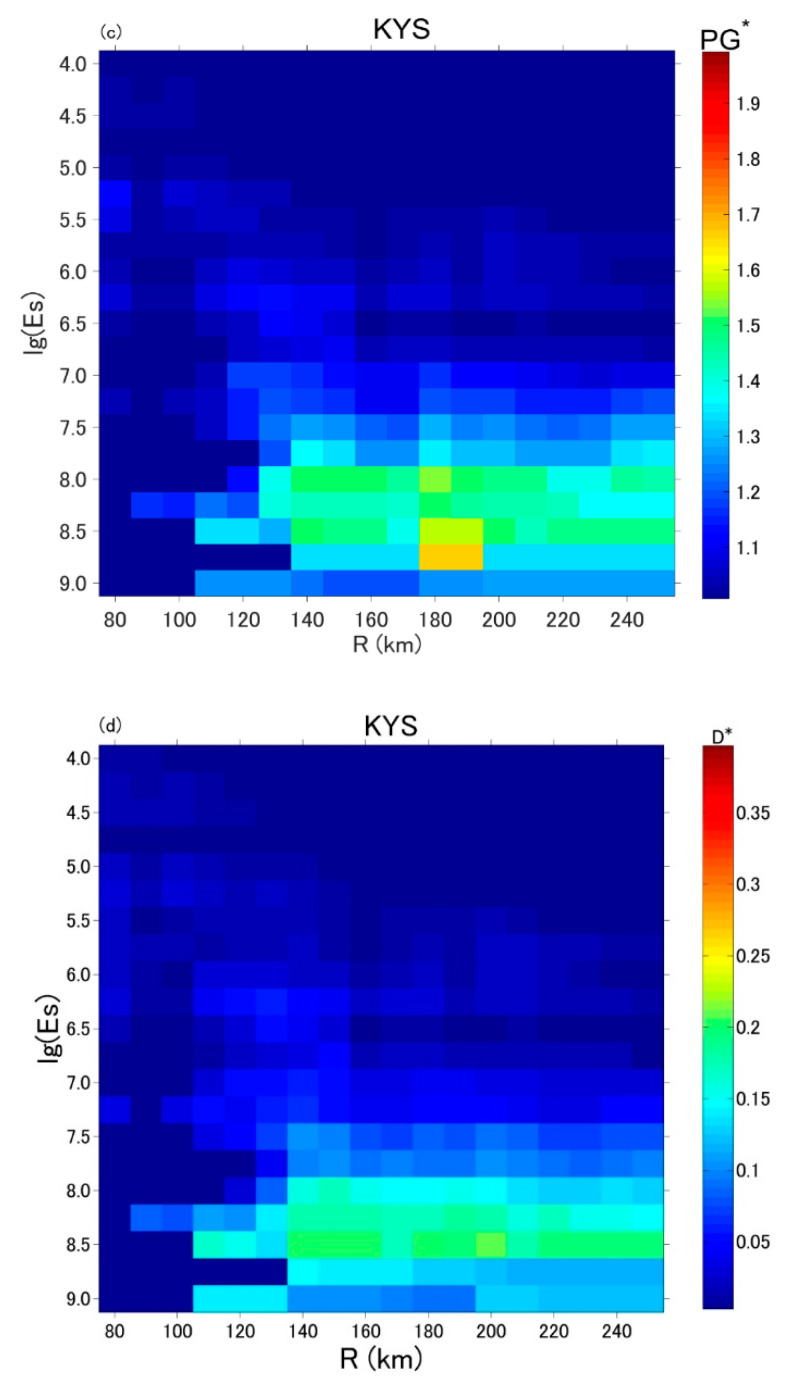
Prediction efficiency using different *R* and *Es*. (**a**) PG* at SKS station; (**b**) D* at SKS station; (**c**) PG* at KYS station; (**d**) D* at KYS station.

First of all, we must quantify the precursory information of each prediction curve. Based on Section 4.3, the *PG′* or *D′* index can help to evaluate the prediction. Therefore, the maximum *PG′* (*D′*) in the curve, namely PG* (D*) is employed as a measure of prediction efficiency. Next, we change *R* from 80 km to 250 km with a step of 10 km and *Es* from 10^4.0^ to 10^9.0^ with an amplifier of 10^0.25^. The ranges of *R* and *Es* are determined by a requirement of earthquake sample number *N* ≥ 4. We plot each prediction curve for different *R* and *Es* parameters and compute the corresponding PG* and D* values. The results are given in Figure 5a,b show the PG* and D* of SKS in Izu, respectively; Figure 5c,d show the PG* and D* of KYS in Boso, respectively. In each panel, the prediction efficiency is shown in color gradation from blue (low value) to red (high value). The results of PG* and D* are quite similar at each station and one could find clear dependence on *Es* and *R* for both SKS and KYS stations. The efficiency gradually increases when *Es* approaches to the value between 10^8.5^ and 10^8.75^ and decreases when it goes higher. This is because with the *Es* threshold going up, the number of irrelevant events that cannot be predicted by magnetic anomalies decreases, till the threshold reaches to the optimal. After that, increasing the *Es* threshold will lead to the loss of relevant events that can create and be predicted by magnetic anomalies. Similar things happen when changing the *R* parameter. Note that sometimes the earthquakes events do not change even using different *R*, and consequently the efficiency may remain the same. In such condition, the optimal *R* should be the minimum one.

**Figure 6:**
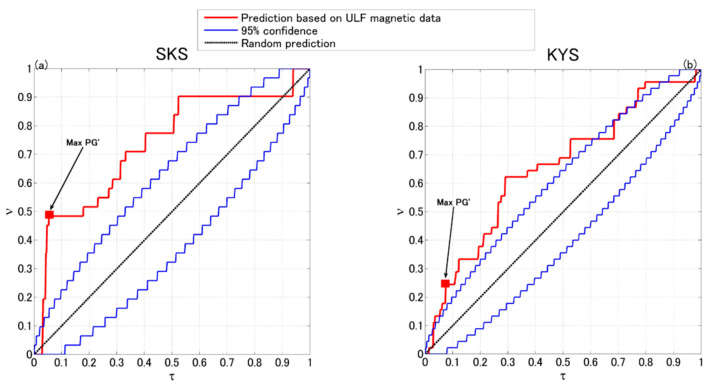
Molchan’s error diagrams of prediction at SKS and KYS. (**a**) The optimal prediction parameters determined by PG*, where *R* = 100 km and *Es* = 10^8.75^ for SKS. (**b**) The optimal prediction parameters determined by PG*, where *R* = 180 km and *Es* = 10^8.75^ for KYS.

In Figure 5, the maximum efficiency suggests the optimal prediction parameters. For SKS station in Izu, both the PG* and D* indices show that the optimal parameters are *R* = 100 km, *Es* = 10^8.75^; for KYS station in Boso, they are *R* = 180 km, *Es* = 10^8.75^ based on PG* and *R* = 200 km, *Es* = 10^8.5^ based on D*. Figure 6 plots the prediction curves using the optimal parameters determined based on PG*. Compared to the predictions in previous study [74], they are clearly more effective in both SKS and KYS stations.

## 6. Discussion

### 6.1. The Influences of Data Missing on Forecasting Performance

**Figure 7:**
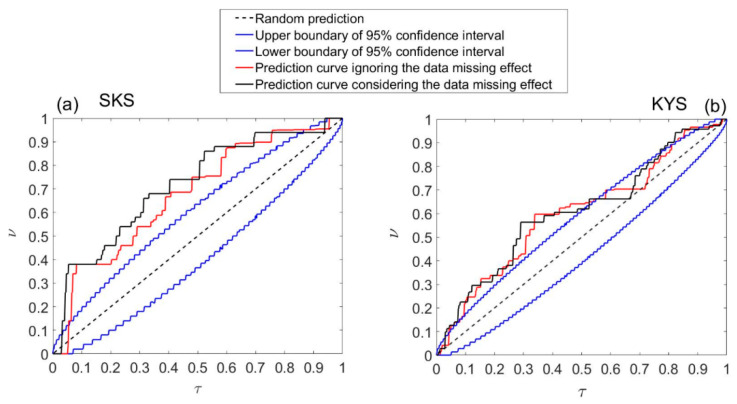
Molchan’s error diagrams for prediction based on magnetic anomalies at SKS and KYS. Diagonal lines in black indicate the prediction performance by the random guess; red solid line shows the prediction curve ignoring the data missing effect; black solid line shows the prediction curve considering the data missing effect; blue curves give the 95% confidence bands of the performance of random predictions. (**a**) Prediction performances with *Es* = 10^8.0^, *R* = 100 km for SKS. (**b**) Prediction performances with *Es* = 10^8.0^, *R* = 150 km for KYS.

As data missing is inevitable in long-term field observations, how to treat the missing data in the alarm algorithm becomes a key problem when assessing the performance of forecast. In order to keep each day getting information equally, we exclude the days affected by data missing in both alarm and event series. Statistically, this is a proper way to treat the missing data. However, in practical, once finding an anomaly on Day *i*, we will alarm the intervals between Day *i* + Δ and Day *i* + Δ + *L* − 1, even though other relevant days in the anomaly series fail to provide alarm information due to data missing. Thus, to assess the practical results, we adopt a new alarm function by ignoring the data missing effect: a day will be alarmed if there is any anomaly between Δ + *L* − 1 day before and Δ day before. Obviously, the new algorithm increases the alarmed days and may also raise predicted earthquake events. Figure 7 plots the prediction curves with and without considering data missing effect together for comparison. As seen from figure, the prediction curves are quite similar. In either case, the prediction performance is better than random guess. The differences between the two at SKS station are mostly resulted from the increase of alarm rate when ignoring data missing effect. All of them are clearly better than random guesses.

### 6.2. Quantification of the Precursory Information

The prediction curve could change significantly in using different parameters. To find out the most effective values, we must quantify the precursory information of each prediction curve. Han et al. [80] adopted the area skill score measuring the area between the actual prediction curve and the random prediction line to search optimal Δ and *L* values. In that study of optimizing time parameters, the earthquake samples were fixed, and the confidence intervals were the same for prediction performances using different Δ and *L*, which enables one to compare the prediction curves directly without taking the confidence intervals into consideration. However, in this study, the number of events varies and the confidence interval changes accordingly when using different *Es* and *R*. Therefore, when quantifying the precursory information, the new indices *PG′* and *D′* which take the influences of confidence intervals into account are introduced. Moreover, these new parameters could indicate whether the precursory information is significant.

### 6.3. Coherence with Other Independent Observations

In previous studies, we have found that the anomaly were more likely to appear a few weeks before the EQs. These results agree with the experimental fact observed in Greece that the lead time of Seismic Electric Signals (SES) activities, which are accompanied by magnetic field variations in the z-component, lies in the range from a few weeks to a few months before reginal sizeable earthquakes [82]. Moreover, the occurrence time for SES was found coincident with the minimum of the order parameter of seismicity [18]. For example, this minimum obtained by applying natural time analysis [83] to the seismicity of Japan was observed in the beginning of January 2011 [82,84], just a few weeks before the M9 Tohoku EQ. The precursory phenomenon was independently confirmed by the simultaneous anomalies in geomagnetic diurnal variations at distances almost 130 km from the Tohoku EQ epicenter, reported by Xu et al. [58] and Han et al. [56,57].

### 6.4. Implications and Applications

To date, although many different kinds of seismoelectromagnetic phenomena in a very wide frequency range have been reported all over the world, none of them can be always detected before all large earthquakes nor always followed by large earthquakes. To address this problem, we have performed statistical studies based on long-term continuous monitoring of ULF magnetic field in seismic areas in Kanto, Japan. The correlation between magnetic anomalies and local seismic events has been verified in our previous study [72]. In this study, we have demonstrated that the magnetic data contain precursory information for sizeable earthquakes and explored their potential capability in improving short-term earthquake forecasts. On the other hand, the feasibility investigation of operational earthquake forecasting based on electromagnetic phenomena has not been conducted. Though many successful case studies have been claimed, most of them are retrospective analysis rather than prospective analysis. Thus, establishing an optimal prediction model (including time, location and magnitude) for prospective forecasting is important and urgent. In this study, we have proposed a method based on Molchan’s error diagram to determine optimal *R* and *Es*, which can be useful in the prediction model. Alternative statistical methods such as Receiver Operating Characteristics (ROC) [85] or Event Coincidence Analysis (ECA) [86] could also be used in future studies to explore optimal forecasting strategy.

There are many studies on earthquake forecast, and most of them adopt catalog-based probabilistic approach [87,88,89,90]. Nevertheless, non-seismological approaches may provide additional useful information and broaden the knowledge of prediction [91]. As stated by Uyeda et al. [92], the approach to the critical state can be clearly identified by analyzing time–series in a newly introduced time domain “natural time”, [93], and thus may shorten the lead–time of SES prediction to only a few days [94]. These latest results imply that seismic data may play an amazing role in short term precursor when combined with SES data. In this study, we have demonstrated another promising candidate of ULF geomagnetic measurement. For the non-seismological observations (e.g., ground water and radon), most of the networks are sparse, which makes it is very different from obtain sensitive distance and size of the earthquake for detecting possible precursory signals. The methodology proposed in this paper may provide a useful way to estimate theses parameters.

## 7. Conclusions

By using Molchan’s error diagram, we have proved that the earthquake predictions based on magnetic anomalies are significantly better than random guess, which suggests that the geomagnetic data recorded in the Kanto Region, Japan contain potential precursory information of local sizable earthquakes. The precursory information exhibits clear *R* and *Es* dependences. To optimize the prediction, we introduced the probability gain (*PG′*) and the probability difference (*D′*) to assess the prediction efficiency. The optimal *R* and *Es* parameters have been explored by searching the maximum efficiency. These results are useful to understand the seismomagnetic phenomena and can improve the operational forecasting model based on ULF electromagnetic approaches. The methodology proposed in this study could also help evaluate the prediction policy and optimize other kinds of measurements for short-term earthquake forecasting.
